# Supplementary material for: Complex‐centric proteome profiling by SEC‐SWATH‐MS
Source: Mol Syst Biol. 2019 Jan 14;15(1):e8438. doi: 10.15252/msb.20188438 (PMC6346213; doi:10.15252/msb.20188438)
Supplement: Supplementary file 8 — Dataset EV7 [file MSB-15-e8438-s008.zip › feature_plots_string/Q99816.pdf]

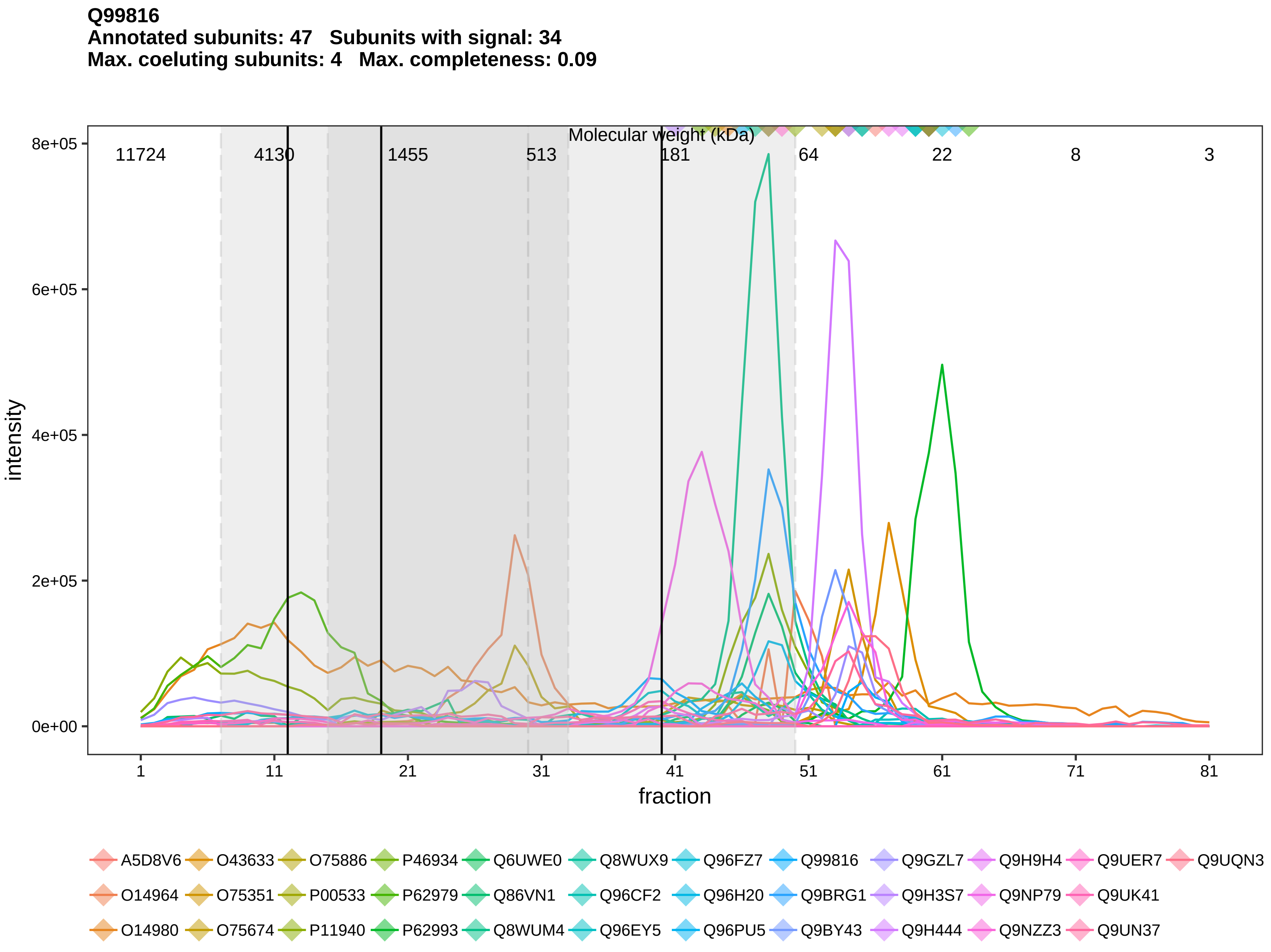
Q99816
Annotated subunits: 47 Subunits with signal: 34
Max. coeluting subunits: 4 Max. completeness: 0.09
Molecular weight (kDa)
11724
4130
1455
513
181
64
22
8
3
8e+05
6e+05
4e+05
2e+05
0e+00
intensity
1
11
21
31
41
51
61
71
81
fraction
A5D8V6
O43633
O75886
P46934
Q6UWE0
Q8WUX9
Q96FZ7
Q99816
Q9GZL7
Q9H9H4
Q9UER7
Q9UQN3
O14964
O75351
P00533
P62979
Q86VN1
Q96CF2
Q96H20
Q9BRG1
Q9H3S7
Q9NP79
Q9UK41
O14980
O75674
P11940
P62993
Q8WUM4
Q96EY5
Q96PU5
Q9BY43
Q9H444
Q9NZZ3
Q9UN37
